# Reversible defect engineering in graphene grain boundaries

**DOI:** 10.1038/s41467-019-09000-8

**Published:** 2019-03-06

**Authors:** Krishna Balasubramanian, Tathagatha Biswas, Priyadarshini Ghosh, Swathi Suran, Abhishek Mishra, Rohan Mishra, Ritesh Sachan, Manish Jain, Manoj Varma, Rudra Pratap, Srinivasan Raghavan

**Affiliations:** 1 Center for Nanoscience and Engineering, Indian Institute of Science, Bangalore, 560012 India; 2 Physics Department, Indian Institute of Science, Bangalore, 560012 India; 3 Materials Research Center, Indian Institute of Science, Bangalore, 560012 India; 4 Department of Mechanical Engineering and Materials Science, Washington University in St. Louis, Washington, MO 63130 USA; 5 Material Science and Technology division, Oak Ridge National Laboratory, Tennessee, 37831 USA; 6 Present Address: Electrical Engineering, Technion Israel Institute of Technology, Haifa, 3200003 Israel; 7 Present Address: Materials Engineering, Technion Israel Institute of Technology, Haifa, 3200003 Israel; 8 Present Address: School of Mechanical and Aerospace Engineering, Oklahoma State University, Stillwater, 74078 OK USA

## Abstract

Research efforts in large area graphene synthesis have been focused on increasing grain size. Here, it is shown that, beyond 1 μm grain size, grain boundary engineering determines the electronic properties of the monolayer. It is established by chemical vapor deposition experiments and first-principle calculations that there is a thermodynamic correlation between the vapor phase chemistry and carbon potential at grain boundaries and triple junctions. As a result, boundary formation can be controlled, and well-formed boundaries can be intentionally made defective, reversibly. In 100 µm long channels this aspect is demonstrated by reversibly changing room temperature electronic mobilities from 1000 to 20,000 cm^2^ V^−1^ s^−1^. Water permeation experiments show that changes are localized to grain boundaries. Electron microscopy is further used to correlate the global vapor phase conditions and the boundary defect types. Such thermodynamic control is essential to enable consistent growth and control of two-dimensional layer properties over large areas.

## Abstract (web summary)

Engineering the grain boundary size of chemical vapor deposited monolayer graphene can reversibly tune its electronic properties. Here, the authors report a thermodynamic correlation for 100 μm long channels in graphene by reversibly changing its electronic mobilities from 1,000 to 20,000 cm^2^ V^−1^ s^−1^.

## Introduction

The chemical vapor deposition (CVD) of graphene for large area applications is typically carried out by transition metal (Cu, Pt, Co, Cr, Rb etc.) catalyzed hydrocarbon (methane, C_2_H_4_, C_6_H_6_ etc.) decomposition at temperatures in excess of 950 °C^1–6^. Continuous monolayers are formed on the metal surface by the nucleation and coalescence of graphene domains. Hence, grain boundaries (GB) and triple junctions that are a part of any polycrystalline material are also present in large area monolayer graphene^7–9^. These GB have been extensively studied and their defect density has been theoretically shown to be mainly dependent on the extent of misorientation between the grains^10–12^. However, in CVD graphene, the defect density at a boundary is not determined by the misorientation angle alone. Tsen et al.^13,14^ studied the effect of source gas flow conditions on the quality and type of GB structures formed in CVD grown graphene. Using transmission electron microscopic (TEM) analysis of the boundary along with electrical transport measurements, they reported that the samples grown at higher methane flows had smaller grains, uniform coverage and most importantly, lower GB resistance. Samples grown using lower methane flows were found to have boundaries with overlaps and voids, resulting in higher resistance^14^. The results are markedly important in two aspects. First, the GB defect density was shown to depend not only on the geometric aspects of the grains such as tilt misorientation and edge type, but also on gas flow conditions. Second, electrical transport characterization was found to be an effective tool to inspect the defect density of GB. Their study, however, did not address the correlation between the physico-chemical state of the growth system and defects at GB. More recently, calculations by Dong et al.^15^ showed that one can toggle between covalently bonded boundaries and hydrogen terminated edges by changing hydrogen pressure.

In this article, it is shown that there exists a thermodynamic relationship between the gas phase and defects in the deposited monolayer. Such defects, which are shown to be predominantly located at the domain coalescence boundaries, can be healed and then re-created, by exposing the monolayer to different corresponding carbon potentials. Hence, monolayer properties can be tuned. As an illustration, it is shown that the charge mobility (*µ*_cm_) and sheet resistance (*R*_s_) can be reversibly changed by more than an order of magnitude. This control has allowed us to synthesize graphene with *µ*_cm_ of 20,000 cm^2^ V^−1^ s^−1^ in device with dimensions of 100 µm × 100 μm. Our results are compared against reported literature in Supplementary Note 1. All these improvements on the monolayer happen in a regime in which Raman measurements^16,17^ are insensitive to the changes occurring in it. Hence, to supplement the electrical transport measurements, water permeation measurement technique is used to identify and spatially locate the incorporated changes in the monolayer. The results highlight the importance of GB closure in obtaining graphene with the best of electronic properties over large areas, in the regime in which the yields of increasing grain size start diminishing (Supplementary Note 2)^18^. In addition, there are many applications that can benefit from the controlled introduction of defects at graphene boundaries. These include the ability to control molecular permeation and sensitivity to various chemicals^19,20^.

## Results

### Nucleation, growth, and annealing at constant Δ*G*

Graphene synthesis using Cu catalyzed dehydrogenation of methane can be described by the following equation.

1 $$\begin{array}{*{20}{c}} {{\mathrm{CH}}_4 \leftrightarrow {\mathrm{C}}_{{\mathrm{Cu}}} + 2{\mathrm{H}}_2} \end{array}$$

**Fig. 1:**
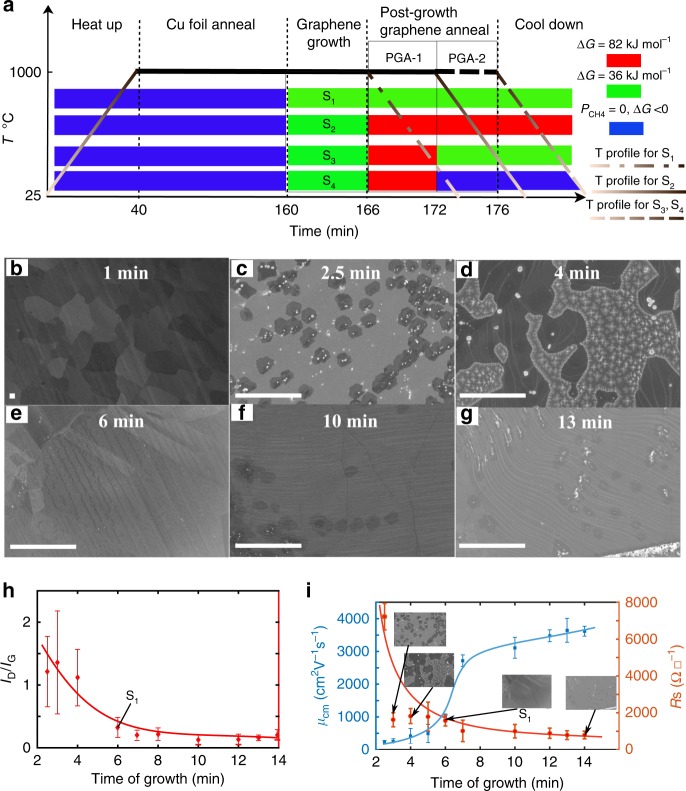
Growth and characteristics of the reference graphene group G_1_. **a** Graphene growth conducted in this work and the steps involved are described schematically. The growth atmosphere is color coded and the legend shows the supersaturation conditions. S_1_–S_4_ refer to four different samples as listed in Table 1. **b**–**g** SEM images of various stages of graphene growth on Cu under conditions used for the standard group G_1_. Growth time (from 160th minute in Fig. 1a) is indicated in the photographs. **b** Incubation period, *t* < 2 min, in which no nuclei are formed. **c** Nucleation stage, *t* = 2.5 min, in which domains of size ~ 2 µm are observed. **d** Growth stage, *t* = 4 min, in which individual domains grow by adatom addition until they merge with other similarly growing domains. **e** Visually coalesced graphene (VCG) observed after 6 min of growth. This sample is referred to as S_1_ and serves as a reference. **f** Surface image after 10 min of growth. **g** Surface image after 14 min of growth. The bright spots are Cu particles that are deposited from the vapor phase during cool down on exposed Cu areas. **h** The time evolution of film defect density as measured by Raman spectroscopy. **i** Charge mobility (*μ*_cm_) and sheet resistance (*R*_s_) evolution with growth time. While *µ*_cm_ shows a four-fold change after visual coalescence at 6 min, Raman defect density remains almost unchanged. A Gompertzian-Sigmoidal fit (blue continuous line in **i**) to the charge mobility is also shown. Scale bars in **b**–**g**: 10 µm

C_Cu_ refers to the carbon monolayer (graphene) on the Cu surface. Equation (1) represents only the thermodynamic terminal points, whereas the internal pathways of the system can be many and are still being debated^2,21^. The free-energy change driving the growth or supersaturation (Δ*G*) is then given by

2 $$\begin{array}{*{20}{c}} {\Delta G = RTln\left( {\frac{K}{{K^{eq}}}} \right)} \end{array}$$

where, $K = P_{{\mathrm{CH}}_4}/P_{{\mathrm{H}}_2}^2$, *K*^*eq*^ is the value *K* at equilibrium, *P*_CH4_, *P*_H2_, *T*, and *R* represent CH_4_ partial pressure, H_2_ partial pressure, temperature, and the gas constant, respectively. Details of the experimental conditions used and the resulting supersaturation, calculated using standard formation energies^22^, are given in the Supplementary Note 3 for a range of methane partial pressures. Four groups of samples, G_1_–G_4_, were studied as summarized in Table 1. The growth details of four specific samples, S_1_–S_4_, one from each of these groups, to be discussed in this paper are presented in Fig. 1a below and also described in Table 1.

**Table 1:** List of sample types and descriptions

Sample group type	Description
G_1_	Standard graphene. Samples grown at a constant Δ*G* of 36 kJ mol^−1^ from 0 and up to 14 min. After 6 min a complete monolayer is observed in the SEM and is called visually coalesced graphene (VCG). This 6-min graphene is sample S_1_. Points corresponding to sample S_1_ are indicated in Fig. 1h, i. Electrical data from sample S_1_ are presented in Supplementary Table 1, row number 5.
G_2_	Annealed graphene. Samples obtained by first growing up to point VCG at 36 kJ mol^−1^, sample S_1_ above, and then exposing to Δ*G* values higher than 36 kJ mol^−1^ in stage PGA-1 in Fig. 1a without breaking vacuum. Points corresponding to the specific sample S_2_ from this group, which was annealed at Δ*G* of 82 kJ mol^−1^, are indicated in Fig. 2a, b. Electrical data from sample S_2_ is presented in Supplementary Table 1, row number 14.
G_3_	Reverse annealed graphene. Samples obtained by first performing growth as in sample S_2_ above and then exposing to Δ*G* values lower than 82 kJ mol^−1^ and down to 36 kJ mol^−1^ in stage PGA-2 in Fig. 1a, without breaking vacuum. Point corresponding to the sample S_3_ from this group, re-annealed at Δ*G* of 36 kJ mol^−1^ is indicated in Fig. 2b. Electrical data from sample S_3_ is presented in Supplementary Table 1, row number number 18.
G_4_	Hydrogen-treated graphene. Samples obtained by first performing growth as in sample S_2_ and then exposing the sample to pure hydrogen ambient, Δ*G* < 0 in PGA-2 without breaking vacuum. Electrical data from sample S_4_ is presented in Supplementary Table 1, row number 19.

In the schematic representation shown in Fig. 1a, a heat-up stage and Cu foil anneal stage are used to set the reactor temperature and anneal the substrate, respectively. Supersaturation, Δ*G* = *G*_reactants_−*G*_products_, is established in the reactor in the growth stage during which sequential methane dehydrogenation produces a population of adatom species on the Cu surface. Graphene growth then proceeds through the four stages of incubation, nucleation, growth, and coalescence. Following the 6-minute point, certain samples, see Table 1, are treated with two kinds of post-growth anneal procedures PGA-1 and PGA-2, without breaking vacuum. Finally, the samples are cooled down under the growth condition to room temperature.

At Δ*G* ≤ 32 kJ mol^−1^, graphene grains either did not nucleate or the rate was so slow that no growing domains were observed until a reasonable growth period of 20 min. Upon increasing the Δ*G* to 36 kJ mol^−1^, group G_1_, the surface remained unmodified up to 1 min as shown in Fig. 1b. Nuclei of size ~2 µm were observed at 2.5 min as shown in Fig. 1c. The individual grains then grew and started to coalesce. Figure 1d shows a partially covered graphene layer after 4 min of growth. Finally, after about 6 min, the Cu surface was “completely covered” as can be seen in Fig. 1e. The graphene film after 6 min of growth will be referred to as “visually coalesced graphene (VCG)” as the SEM micrographs indicate complete coverage and thereafter no further change is detected by SEM. This is sample S_1_. Surface images of Cu growth surface at 10 mins and 13 mins are shown in figure 1f, g, respectively. The average film defect density extracted from Raman spectroscopic measurements, presented in figure 1h, (see Supplementary Note 4 for Raman measurement details), also mirrors this temporal evolution. The I_D_/I_G_ ratio decreases sharply upto a growth time of 6 min and then saturates in the period from 6 to 14 min. Thus, it appears from these characterization methods that monolayer deposition is complete by 6 min. Graphene growth by CVD on Cu is typically modeled as a self-limiting catalytic process^4^, which can explain the SEM snapshots and I_D_/I_G_ saturation behavior. However, as can be noted from Fig. 1i, *µ*_cm_ increases rapidly only after 6 min of growth—the point at which the monolayer seems complete—and saturates at 13 min. The *µ*_cm_ here is obtained from the constant mobility fit described by Venugopal et al.^23^ as explained in Supplementary Notes 5 and 6. Kinetic growth saturation has been previously observed in graphene and the properties were shown to have a Gompertzian-sigmoidal behavior^24^. A Gompertzian fit, shows that *µ*_cm_ increases by a factor of 4 from 1000 cm^2 ^V^−1 ^s^−1^ at 6 min to saturate at 3600 cm^2 ^V^−1 ^s^−1^ at 14 min. Sheet resistance decreases by a factor of 2 and attains a saturation value of 790 Ω☐^−1^ at 14 min. These observations belong to the sample set G_1_ in which a constant *ΔG* of 36 kJ mol^−1^ was maintained through all stages of growth. This sharp change in electronic properties, beyond the point at which the other structural characterization methods and Raman defect density measurements do not suggest any significant change in the quality of graphene is the first interesting observation. All the electrical measurements, except for samples grown below 4 min (where the film was not completely coalesced), were performed on 100 μm by 100 μm graphene devices (See Supplementary Notes 5–7, for more details regarding electrical measurements).

### Tuning GB and electron mobility by varying Δ*G*

Further experiments, sample group G_2_ in Table 1, were conducted to determine whether *µ*_cm_ and sheet resistance can be further improved with the grain size unchanged. Graphene layers were deposited with Δ*G* conditions kept the same as in sample S_1_ in the growth stage (see Fig. 1a). This ensured that the average graphene grain sizes were constant across all samples in this group. Post the VCG stage, these samples were annealed without breaking vacuum for 6 min with higher Δ*G* values up to 112 kJ mol^−1^. A different growth run was used for each value of Δ*G* used for annealing (see Supplementary Table 1). This is denoted as PGA-1 in Fig. 1a. At higher ∆*G*, growth transients were expected to attain the final steady states of interest within 6 min. Hence, a time sequence such as the one in Fig. 1i was not performed for these higher Δ*G* values.

**Fig. 2:**
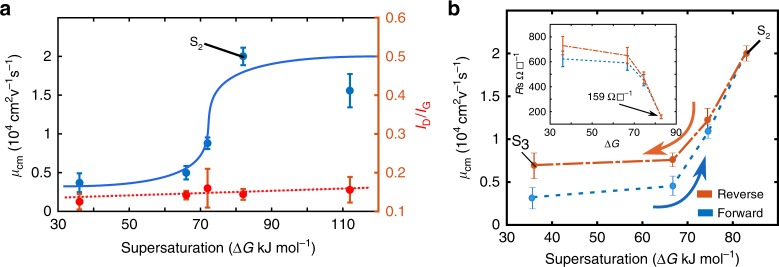
Reversibility in mobility with reversal in thermodynamic conditions. **a** Effect of annealing sample S_1_ under increasing supersaturations (∆*G*) on mobility (*µ*_cm_) and Raman I_D_/I_G_ ratio. *µ*_cm_ is seen to increase significantly with Δ*G* and then saturate. The averaged defect density as measured by Raman is insensitive to this change. The sample S_2_ (Δ*G* = 82 kJ mol^−1^) is indicated. **b** Reversal of *µ*_cm_ on reversal of supersaturation, indicating that the phenomenon is thermodynamic in origin and not kinetic. The small hysteresis can be attributed to kinetics. The sheet resistance vs supersaturation and resistance vs gate bias plots are included in the Supplementary Note 6. Samples S_2_ and S_3_ are indicated

The rather dramatic effects of increasing Δ*G* on the saturation *µ*_cm_ are shown in Fig. 2a and sheet resistance is shown in Supplementary Note 6. The charge mobility *µ*_cm_ increases by 20-fold from 1000 to 20,000 cm^2^ V^−1^ s^−1^. The standard deviation in the observed values was < 10% of the mean values and is shown in the Fig. 2a. Correspondingly, there is a further five-fold reduction in the steady state sheet resistance value to 159 Ω☐^−1^ on raising Δ*G* from 36 to 82 kJ mol^−1^. This is the lowest reported sheet resistance thus far (see Supplementary Note 1 and 2 for a compilation of sheet resistances and *µ*_cm_ reported in the literature) in large area CVD graphene and over such large device sizes (100 µm × 100 µm). The Raman I_D_/I_G_ ratio again remains flat. Thus, from the VCG stage at which the structural characterization methods seem to have become insensitive to changes in the graphene layer, further processing has thus resulted in a 20-fold increase in *µ*_cm_. This value is also five-fold higher than the saturation mobility observed under a constant Δ*G* = 36 kJ mol^−1^ in G_1_ (Fig. 1i). The range of sheet resistances achieved here surpass the values obtained in the literature and its comparison is presented in Supplementary Figure 2a.

Additional experiments were performed to determine whether the rise in mobility in Fig. 2a is reversible. Graphene layers were deposited under conditions that yielded the highest *µ*_cm_ and lowest sheet resistance values (after a treatment under Δ*G* = 82 kJ mol^−1^), sample S_2_. Without breaking vacuum these samples were now exposed to decreasing values of Δ*G* down to 36 kJ mol^−1^ for 4 min and 12 min. A different growth run was used for each value of lower Δ*G* used during the reversal. It was observed that the difference in *µ*_cm_ values between the 4-min exposure and the 12-min exposure was within experimental error. Hence, all subsequent exposures to lower Δ*G* were limited to 4 min. The results of these measurements are presented in Fig. 2b. Indeed, the *µ*_cm_ values decrease as Δ*G* is lowered in the reactor and trace back the preceding rise almost exactly but for a small hysteresis.

## Discussion

An atomistic picture of the nucleation, growth, and coalescence process of graphene is now discussed to help analyze these mobility results and the ones to be discussed in the next section. It is shown that the reversible phenomena is due to grain boundary thermodynamics and not owing to growth kinetics. From a growth perspective, it is important to establish this fact, as thermodynamic control is more robust than a kinetic one.

**Fig. 3:**
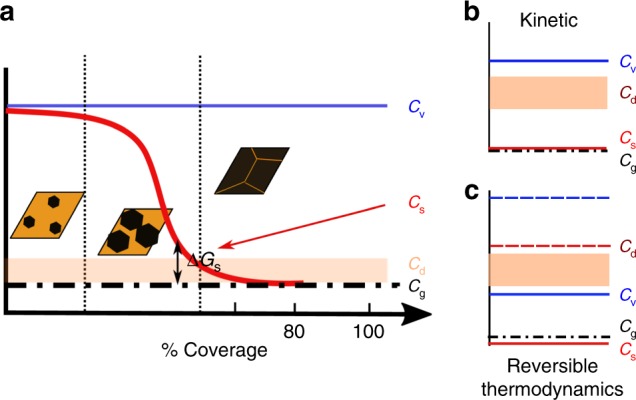
Schematic of growth thermodynamics and kinetics. **a** Carbon potentials at various coverage levels with reference to that inside the graphene grain *C*_g_. *C*_d_, *C*_s_, and *C*_v_ represent defect (at grain boundary), surface and vapor phase potentials. **b** Energetic situation in which the lack of grain boundary closure will be termed kinetic. Vapor carbon potentials are greater than those at the defect levels and thus closure is not impeded by global thermodynamics. However, local (in the vicinity of the grain boundary being formed) surface potentials determined by adsorption-desorption kinetics can be lower than the defect potential thus preventing closure. **c** Energetic situation in which the lack of grain boundary closure is thermodynamic. Those grain boundary structures in which the carbon potentials are higher than *C*_v_ cannot be formed. Raising carbon potentials will form these structures, as shown by dotted lines, whereas lowering it will remove carbon from these locations leaving behind vacant sites

The atomistic picture is shown schematically in Fig. 3a. We use the potential of carbon atoms at various locations in the growth environment to first qualitatively explain the observed phenomena. We later use density functional theory (DFT) calculations to support these conclusions. The graphene growth reaction starts by setting a carbon potential in the vapor phase (*C*_v_) in the form of the methane partial pressure. Methane decomposes on the catalyst and yields a surface carbon potential (*C*_s_). Crystalline graphene domains with a potential *C*_g_ (carbon potential within the graphene grain) nucleate and grow by consuming the surface carbon. *C*_s_ at any point on the growth surface will vary with time and will be determined by the balance between carbon addition by adsorption and decomposition and carbon removal by desorption, nucleation, and attachment to growing edges. With an increase in coverage, as the domains get closer to form boundaries, the *C*_s_ values will get lowered due to reduced catalyst site availability and increased consumption. The reduced *C*_s_ will lower lateral growth rates and in the extreme case prevent complete coalescence in reasonable time periods. In addition to these three potentials, carbon atoms in the defect structures such as GB, triple junction and line defects in the graphene monolayer will be at higher carbon potentials (*C*_d_) than *C*_g_ and are shown in Fig. 3a as a band.

If *C*_s_ became equal to *C*_g_ during growth, a coverage saturation will happen despite a global supersaturation (*C*_v_ being higher than *C*_g_). If *C*_s_ were raised by increasing *C*_v_ (increasing *ΔG*), the degree of coverage will increase in a given time of growth. Such growth is termed kinetically controlled and the coverage saturation owing to kinetic limitations has been described before by Celibi et al. using a Gompertzian model^24^. Thus, the increase in mobility with Δ*G* or methane partial pressures in Fig. 2a can be attributed to “more faster and complete coverage” in the time frames involved, in the sense that the grain boundary closure is more complete. If this were true, that is, the improvements in mobility in Fig. 2a were purely owing to kinetic reasons as just discussed, then re-annealing the samples that had attained the highest mobilities in lower methane partial pressures (*C*_v_ still higher than *C*_g_) should not reverse the mobility gains as observed in Fig. 2b. The reversal is thus proof of the improvements, which happen post grain coalescence, having a thermodynamic origin.

Thermodynamically, the ability to form a particular boundary structure that will complete the coalescence process, will require the global carbon potential, *C*_v_ to be greater than *C*_d_ as shown in Fig. 3b. If in spite of this scenario it did not form, then the effect is kinetic. Such a situation will arise if *C*_s_ determined by the relative rates of adsorption–attachment and desorption were to become lesser than *C*_d_. On the other hand, if the situation shown in Fig. 3b prevailed, then, thermodynamic constraints will come into play. Those boundary structures that have *C*_d_>*C*_v_ will never form as it will not be energetically favorable for carbon from the vapor phase to become part of these defect structures. Increasing/decreasing *C*_v_ will then add/remove carbon from these structures, thus healing/forming defects, vacant carbon sites, reversibly at these locations. The spatial and temporal variation of *C*_s_ is difficult to estimate quantitatively. Hence, in the next section we compare the energetics of *C*_v_, with that of boundary energies to determine if such an effect is possible.

Dong et al.^15^ have shown that by changing the hydrogen potential in the reactor, one can replace covalently bonded boundaries by hydrogen terminated ones and vice-versa. The experimental work presented here involves changing the carbon potential in the reactor resulting in reversible exchange of carbon atoms between the boundary and the vapor phase. To model this process (See Supplementary Note 8 for the thermochemistry considerations) density functional simulations were carried out to compare the energetic differences between different states of the grain and its boundaries. The thermodynamic reversibility at the grain boundary implies that there are two states, a grain boundary and a defective grain boundary, between which the system can toggle depending on the carbon potential in the system. The defective grain boundary will have to be formed by the breaking of carbon–carbon bonds. It is expected that the resulting dangling bonds will get passivated by hydrogen in the hydrocarbon environment of the reactor. This resultant entity can be approximated by a graphene edge terminated by hydrogen. The reaction that represents this process can be expressed as:

3 $$\begin{array}{*{20}{c}} {\mathrm{C} - {\mathrm{C}}^\prime - \mathrm{C} + 3{\mathrm{H}}_2 \leftrightarrow 2 \mathrm{C} - \mathrm{H} + {\mathrm{CH}}_4} \end{array}$$

**Fig. 4:**
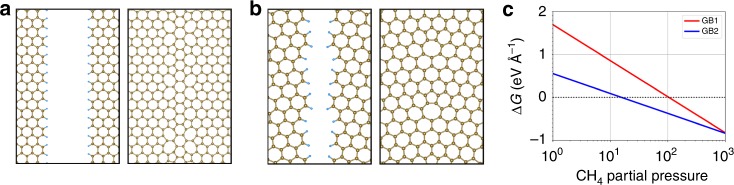
DFT simulation of graphene grain boundary energetics with methane partial pressure. **a**, **b** represent two random mis-orientations GB1 and GB2, respectively. For both, the grain edge passivated with hydrogen, blue atoms, and defect structure on boundary formation are shown. **c** The free energy change, *ΔG*, for these two configurations as per equation (3) vs methane partial pressure. A crossover in the plot suggests that depending on the methane partial pressure the reaction can proceed in the forward or backward direction. Increase in pressure favors boundary formation and vice-versa as observed experimentally. The pressure at which it happens depends on the boundary misorientation

“C′” represents grain boundary carbon and the equation shows a defect removal and its replacement by an edge. In order to determine whether an edge state with lower energy can exist, two graphene grain boundary configurations and their corresponding edges, Fig. 4a, b, were chosen. DFT calculations were performed (see details in Supplementary Note 8) to determine the energies of the grain boundary and the edge. Data from JANAF tables were used to calculate the potentials of hydrogen from H_2_ and carbon from CH_4_. These results were then used to calculate the free energy change for the reaction in equation (3) (Δ*G* = *G*_Reactants_−*G*_Products_). The *ΔG* variation with methane partial pressure is plotted in Fig. 4c.

A positive value means that the reaction can proceed in the forward direction, i.e., an edge can replace a boundary as it has lower energy. A negative value means that the boundary will be stable against such breakup and defect creation. The plot shows that indeed, a pressure range exists, in which the edge has lower energy than the boundary. There is a crossover after which the reverse is true. The crossover pressure is dependent on the grain boundary configuration. Thus, if at the temperature of graphene growth, there are boundary configurations with positive *ΔG*, then the phenomenon observed will occur at these sites. This will then result in the thermodynamic reversibility observed.

**Fig. 5:**
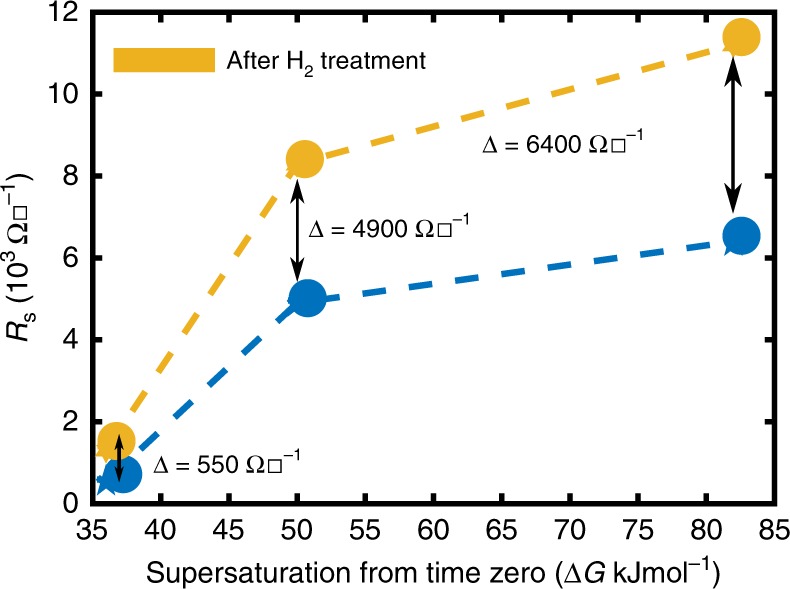
Effect of grain size on sheet resistance change due to hydrogen treatment. *R*_s_ of films nucleated and grown at different supersaturations (See Supplementary Note 9 for effect of supersaturation on grain size) are shown in blue. *R*_s_ obtained after a pure hydrogen anneal, or the lowest carbon potential exposure possible, are shown in yellow. Changes in sheet resistance are also marked. Sheet resistance and its change increase with a decrease in grain size, thereby proving that the responsible defects lie at the GB

In the previous discussion, we had hypothesized that vacant carbon sites can be annihilated and created reversibly at GB causing the mobility reversal with change in vapor phase carbon potential. First principles calculations supported this idea. In this section we provide experimental proof. A simple experiment was first performed in which graphene samples with decreasing grain sizes, obtained by increasing supersaturation in the nucleation stage itself (see Supplementary Note 9), were exposed to 5 min of pure hydrogen at a pressure of 400 Torr and 1000 °C. The resulting change in the sheet resistance before and after hydrogen treatment is shown in Fig. 5. It is clearly seen that under the same hydrogen treatment, which is expected to create defects in the graphene film, samples grown under higher Δ*G* and hence smaller grain sizes (or larger grain boundary length per unit area) not only have a larger sheet resistance but also the highest increase on hydrogen annealing. This indicates that the defects are predominantly created at the GB.

**Fig. 6:**
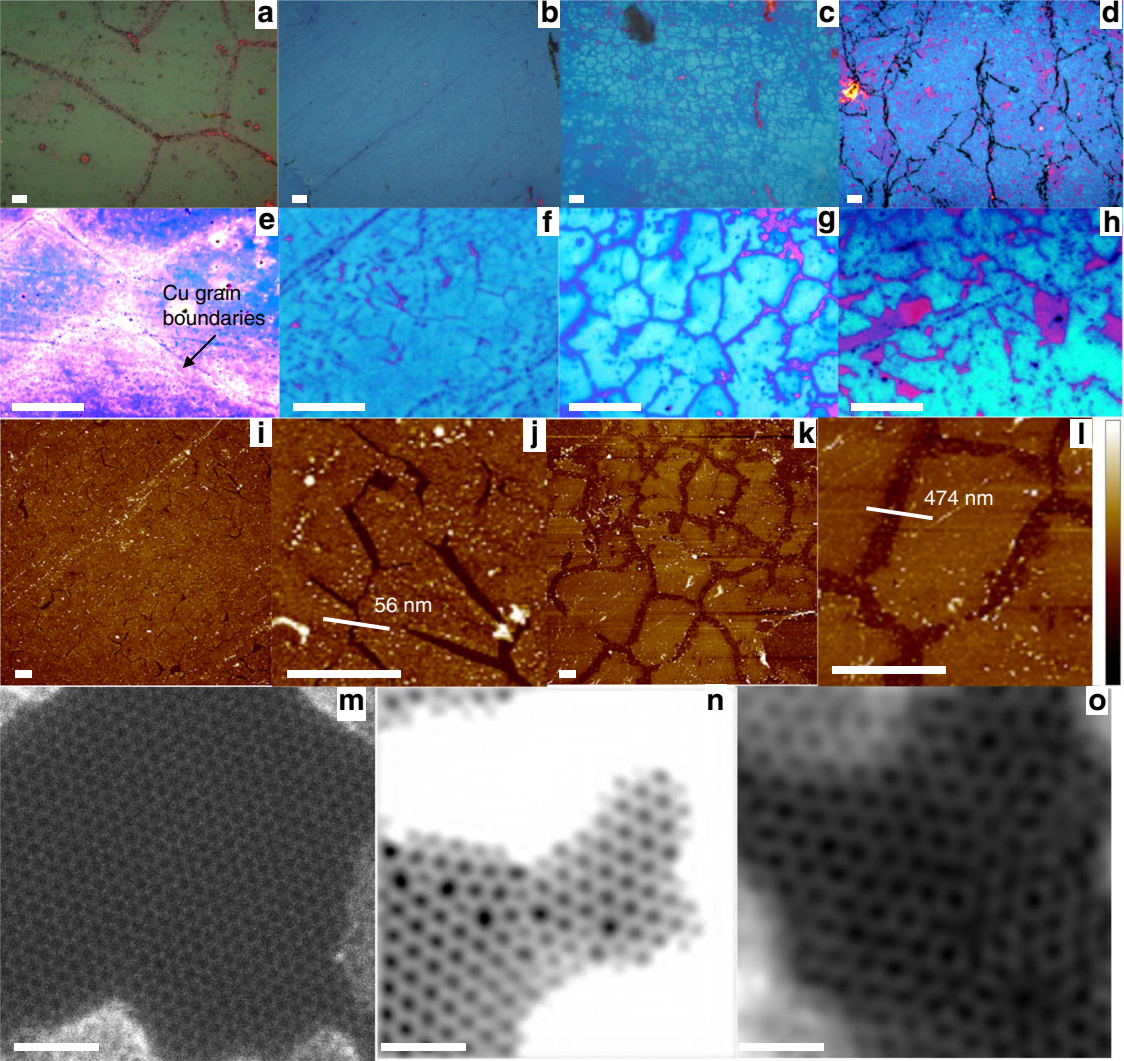
Visualizing defects. Optical images of Ge underlayer etched by water percolating through defects in the graphene overlayer. **a**–**h** Show results after the water percolation experiment on samples S_1–4_ as described in the text. **a**–**d** show the 5× images of the samples S_1–4_, whereas **e**–**h** show a higher magnification (50 ×) of the same sequence. Although **a**–**d** show large features such as Cu GBs, graphene GBs are clearly visible in **f**–**h**. In sample S_1_, visually coalesced graphene, the predominant defects lie at the GBs of the Cu foil when it is annealed for 2 h. Grains are 50–100 µm in size. In all other cases the defects lie at the coalescence boundaries, 5–10 µm apart, of the graphene monolayer. GBs in S_2_ annealed at higher supersaturation, are less defective than in S_3_, as indicated by the higher water percolation rates and hence more deeply etched boundaries. Even annealing in pure hydrogen, S_4_, only results in defects at the boundary and not within the grain. The purple patches in **a**–**h** correspond to graphene grains that have flaked off. The pink dots in **h** correspond to triple junctions. **i** 40 micron square AFM scan of surface of S_2_ after the percolation experiment. **j** 7 micron square AFM scan of surface of S_2_ after permeation experiment with line scan to show etch pit width. **k** 40 micron square AFM scan of surface of S_3_ after the percolation experiment. **l** 7 micron square AFM scan of surface of S_3_ after percolation experiment with line scan to show etch width. **m** Shows a pristine graphene lattice without defects in S_1_. **n** Shows a representative boundary in sample S_1_ with a tilt misorientation of 13.86°. **o** Representative boundary in sample S_4_ with a misorientation of 18°. Image contrast adjusted for better visibility. Scale bars: **a**–**h** 10 μm; **i**–**l:** 3 μm and **m**–**o:** 1 nm. Color bar for **i**–**l:** − 20 nm to 20 nm

In all the four samples previously discussed, S_1_–S_4_, it was noted that while *µ*_cm_ showed variations, Raman defect density remained mostly unaltered. As Raman defect density is an average value obtained from a large area scan (10 µm × 10 µm), the changes should be happening at length scales that the mapped average does not reflect. To understand the physical picture behind the reversible defect density-mobility trend, a method involving preferential water permeation through defects in graphene followed by etching of an underlying Ge film^25,26^ was employed on the four samples listed in Table 1. This process creates a replica of the defect structure in the graphene monolayer on the underlying Ge film and renders them optically visible. A detailed time sequence of the etching to be discussed in the following section is presented in the Supplementary Note 10. Optical images are presented in Fig. 6a–h and AFM images in Fig. 6i–l. Figure 6a, e show that when the VCG (sample S_1_) is examined by this method, the etch pattern formed within just 1 min of permeation, involving 50–100 µm sized grains, corresponds to the microstructure of the Cu substrate on which the monolayer was synthesized (see Fig. 1b). Thus, most of the major defects in VCG lie at the Cu GB. In contrast, in S_2_, S_3_, and S_4_, samples that have been annealed in methane post VCG, the Cu GB are not revealed even after 60 min of permeation (Fig. 6b–d, f–h). Higher magnification images, second row, show that the etch pattern has an average grain size of 6.7 μm (standard deviation of 0.8 μm), which matches well with the values measured using SEM (see Supplementary Note 2). Thus, in these samples the defective regions are predominantly at the coalescence boundaries in the graphene layer.

In addition, in the reverse annealed sample S_3_, the etch pattern lines are thicker (Fig. 6c, g) compared to the annealed sample S_2_, (Fig. 6b, f). The AFM scans shown in Fig. 6(i–l) reveal that difference in the surface after permeation experiments in sample S_2_ and S_3_. Larger etch pit widths quantitatively correspond to higher rates of permeation^25^ enabled by more defective boundaries. After 100 min etch, the wider pit depth of 474 nm in the reverse annealed sample S_3_, when compared to 56 nm in S_2_, clearly shows that reverse annealing had indeed made the GB of the monolayer more defective. Further details on the AFM scans are shown in the Supplementary Note 11. Thus, reverse annealing at lower Δ*G* has made the graphene GB more defective. One can argue that the reverse annealing might also cause defects within the grain (HRTEM imaging in the next section throws more light on this aspect) that are not detected by the permeability measurements. To determine if this is so, sample S_4_ was annealed in pure hydrogen at a pressure of 4 Torr. Even in this case as can be seen from Fig. 6d, h only GB are revealed. If the grain were to have become defective, then one will see more uniform etching of the Ge below. Thus, these results are direct evidence for our thesis that the reversible rise and fall in *µ*_cm_, Fig. 2b, is owing to a decrease/increase in grain boundary defects with a rise/fall in ∆*G*. The type of defects (structure) and their density along the GB of different samples were inspected using a high resolution TEM. Imaging in a 200 kV TEM was first performed over large areas to establish that the graphene being studied was indeed predominantly a monolayer using tilt diffraction measurements (see Supplementary Note 12). The samples were then observed with an aberration corrected scanning transmission electron microscope (STEM) under a 60 kV electron beam. A large pristine region of the graphene film in S_1_ is shown in Fig. 6m. The figure shows that the bulk of graphene is mostly defect free with negligible point or line defect density and that the imaging process does not create any noticeable damage to the lattice. Figure 6n shows a representative grain boundary in S_1_ and Fig. 6o shows a representative boundary in S_4_. While the boundary in Fig. 6n shows a string of Stone–Wales defect structures distributed in a region less than 1 nm, that in Fig. 6o shows a region that is at least 2 nm wide. The grains in Fig. 6n have a misorientation angle of 13.8° and the defective atomic columns consist of continuous Stone–Wales (5–7) structures (see Supplementary Note 13 and 14 for details on angular misorientation calculations and hexagon to Stone–Wales ratio calculations). The ratio of these structures to those of the standard hexagon is > 1. Theoretical predictions based on geometric considerations (Supplementary Note 14) predict a ratio of < 0.6 for a misorientation of ~ 13.8^10^. Thus, the higher defect density at the boundary in S_1_, the 6 min VCG, is indicative of a graphene that has not been fully formed. The triple junction that is close to the imaged boundary can also influence the defect density. However, what this simple calculation nevertheless shows is that defect densities at boundaries and especially closer to triple junctions will be much larger than those theoretically anticipated. They in turn can have a significant impact on the properties of the monolayer. On the other hand, Fig. 6o shows a boundary, which is both wider and higher in defect density than the one in Fig. 6n. Voids are seen at the boundary in addition to standard Stone–Wales defect sites clearly showing the effect of annealing at lower ∆*G*. This explains the lower *µ*_cm_ (see Supplementary Table 1) and high water permeability in S_4_. Only covalently bonded GB were observed at the supersaturations used in the work done as part of this research as can be noted from the SEM and STEM images. Overlapped boundaries as reported by other groups^15^ were not observed under the conditions used for synthesizing the samples used in this paper (Supplementary Note 3). However, at much lower methane partial pressures and lower growth rates, overlapped GB were also observed. A discussion of the characteristics of those samples is beyond the scope of this paper.

In summary, we show that the configuration of grain boundary defects in graphene monolayers have a thermodynamic relationship with carbon potential in the vapor phase. These defects can be created and healed reversibly. This relationship allows one to control grain boundary structure. The resulting large variation in grain boundary resistance allows the field effect mobility in graphene monolayers to be changed by more than an order of magnitude, from 1000 cm^2 ^V^−1 ^s^−1^ to 20,000 cm^2 ^V^−1 ^s^−1^ and sheet resistance from 1200 to 159 Ω☐^-1^, the best reported yet for such large device sizes. Most of these microstructural changes that result in such large variations in electrical properties cannot be detected by standard Raman and SEM characterization. The study also shows that GB might be equally, if not more important, than grain size in graphene.

## Methods

### CVD graphene growth

Graphene was grown in a custom-made CVD reactor^22,27^. Ultra-pure (99.9999%) hydrogen, and nitrogen flowing through a purifier, 99.98% pure methane and 99.98% pure Cu from Sigma Aldrich were used for synthesis. Although growth was done at 1000 °C, reactor chemistry was changed to vary carbon potential by varying methane and hydrogen flows. Gas flows for the supersaturation used in this article are shown in Supplementary Fig. 3. Total reactor pressure was maintained at 4 Torr for all our experiments. Such a condition was chosen to have >85% monolayer coverage. A wet transfer process as described elsewhere^27^ was used to transfer graphene onto a 285 nm thermally grown silica/heavily doped *p*-type Si substrate.

Each data point in Figs. 1i, 2a, b, and 5 were generated by measurements on monolayers grown for that specific purpose without breaking vacuum from start to finish as explained in the text. The standard deviations were calculated from measurements made on at least four devices from a growth run. The growth experiments were also repeated to check the result consistency. The information about the samples and their details are provided in the list of samples provided in Supplementary Table 1.

### Raman measurements

Raman spectroscopic measurements on these transferred layers were carried out with a LabRAM HR spectrometer fitted with a 532 nm laser source. The instrument was fitted with precision micrometer stage for mapped measurements. Further details on Raman spectroscopic measurements are provided in Supplementary Note 4.

### Electrical measurements

Van der Pauw devices for sheet resistance measurements and MOS FET structures for mobility measurements were fabricated using standard optical lithographic techniques and the graphene channel was defined using a mild oxygen plasma etch using an Oxford reactive ion etcher. Except for electrical measurements below 4 min in Fig. 1i all the electrical measurements were made on 100 µm × 100 µm area as shown in Supplementary Note 5. The measurement statistics and fitting examples are also provided in Supplementary Note 6. For the samples grown for less than 4 min, the graphene film was not continuous and hence, electron beam lithographically made devices were used. Details are provided in Supplementary Note 7. All electrical measurements were made after current annealing the sample under a current density of 3 × 10^8^ A/cm^2^ for 3 min under a pressure of 4 × 10^−5^ mbar of air. Given that the average grain size is ~ 7.08 μm (standard deviation of 0.88 μm), (See Supplementary Fig. 2) the 100 μm channel widths are expected to yield average values that represent electronic properties over large areas.

### Etch measurements and AFM

Ge etch experiments to characterize defects, developed in our group^25^, were conducted using an Olympus made immersion lens setup fitted with an Olympus optical acquisition system at room temperature and in open air. The etch profile measurements^25^ were performed using a Bruker made atomic force microscope.

### TEM

Diffraction measurements were performed using a FEI T20 transmission electron microscope at 200 kV and the STEM measurements were made using an aberration corrected Nion Ultra STEM 100 microscope operated at 60 KV.

## Supplementary information

Supplementary Information

## Data Availability

The data that support the findings of this study are available from the corresponding author upon reasonable request.
