# Supplementary material for: Reversible defect engineering in graphene grain boundaries
Source: Nat Commun. 2019 Mar 6;10:1090. doi: 10.1038/s41467-019-09000-8 (PMC6403358; doi:10.1038/s41467-019-09000-8)
Supplement: Supplementary file 1 — Supplementary Information [file 41467_2019_9000_MOESM1_ESM.pdf]

Reversible Defect Engineering in Graphene Grain Boundaries Supplementary Information

Krishna Balasubramanian[1*], Tathagatha Biswas[2], Priyadarshini Ghosh[1,3†], Swathi Suran[1], Abhishek Mishra[1], Rohan Mishra[4], Ritesh Sachan[5‡], Manish Jain[2], Manoj Varma[1], Rudra Pratap[1], and Srinivasan Raghavan[1§]
[1]Center for Nanoscience and Engineering, Indian Institute of Science, Bangalore, India
[2] Physics Department, Indian Institute of Science, Bangalore, India
[3]Materials Research Center, Indian Institute of Science, Bangalore, India
[4]Department of Mechanical Engineering and Materials Science, Washington University in St. Louis, MO 63130, United States
[5]Material science and Technology division, Oak Ridge National Laboratory, Tennessee 37831, United States

[*] Currently at Electrical Engineering, Technion Israel Institute of Technology, Israel
[†] Currently at Materials Engineering, Technion Israel Institute of Technology, Israel
[‡] Currently at School of Mechanical and Aerospace Engineering, Oklahoma State University, United States
[§] Corresponding author. sraghavan@iisc.ac.in

Supplementary Note 1 Literature summary of reported carrier mobilities and the corresponding channel dimensions

The literature on graphene field effect devices is vast and dates back to 2004. There have been several reports of extraordinarily high charge mobilities in graphene. The reported values vary depending on type of graphene, substrate used, encapsulation provided, temperature of measurement, method of measurement and very importantly device dimensions. In Supplementary Figure 1, data from the literature has been summarized. Values obtained in this work have also been included for comparison. It is easily seen that the combination of the range of mobility values demonstrated in this work and the channel size used for measurement is the best thus far. For a 100 μm channel size, the room temperature mobility of 20000 $cm^2V^{-1}s^{-1}$ is the largest value reported value yet, to the best of our knowledge. In the case of CVD grown graphene and other large area practical applications, charge mobility at room temperature and over large areas, and hence such larger channel dimensions, are of importance.

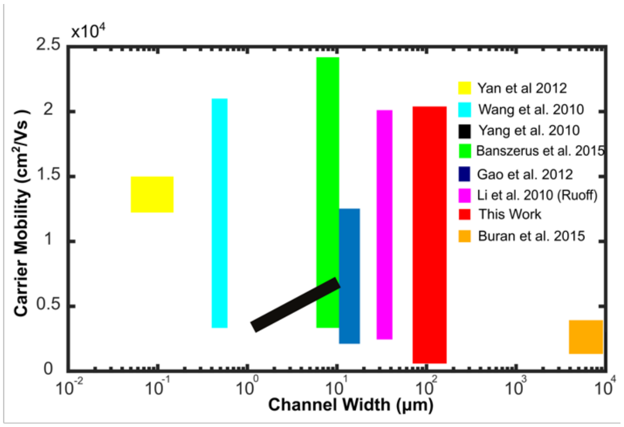

Supplementary Figure 1: Literature summary of room temperature electronic mobility in monolayer graphene and the device channel dimensions used for the measurements. [1, 2, 3, 4, 5, 6, 7, 8] For a channel dimension of 100 μm – one of the largest used and one that is therefore representative of the large area characteristics of the monolayer- the range of mobilities measured and the largest mobility observed in this study are the best thus far.

Supplementary Note 2 Graphene grain boundary resistivity calculations

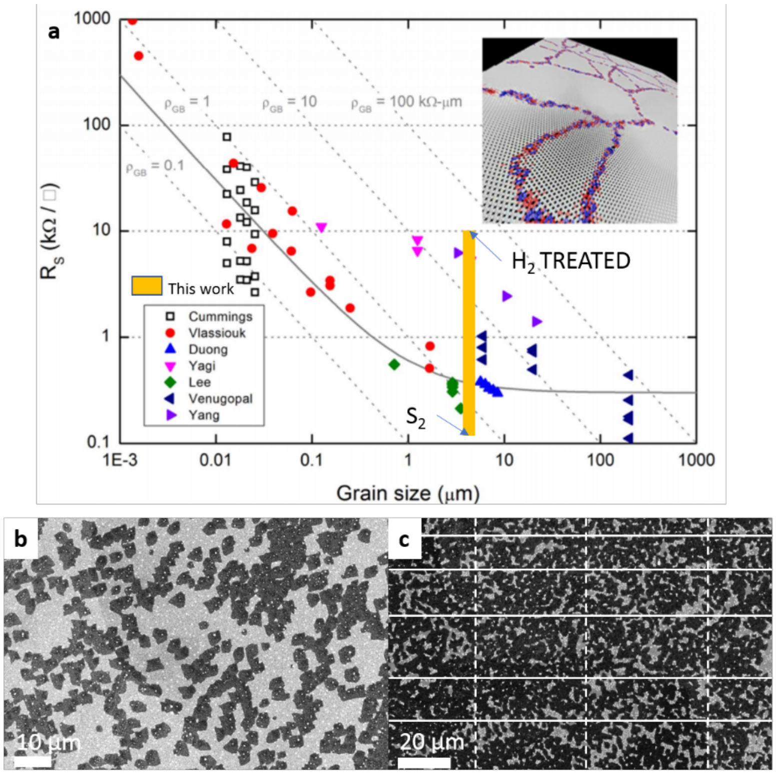

Supplementary Figure 2: Grain size information. **a** The range of sheet resistance measured in this work is compared with literature data as summarized recently by Isacsson et al. [9] It can be seen that range reported here is larger than that anticipated from current literature trends. Image reproduced from IOP Science. Under Creative Commons. **b** Image of uncoalesced islands at 2.5 minutes. **c** Image of the uncoalesced islands just before complete coalescence at 4 minutes. The average grain size was estimated from counting the number of edges crossing the lines shown.

Since graphene grain boundaries are the most defective regions in graphene, they are expected to contribute the most to the film sheet resistance. The reported comparison of measured GB resistance is shown in Supplementary Figure 2**a**. From sheet resistance measurements, one can extract the grain boundary resistivity using the Supplementary Equation 1 given by Isacsson et al. [9]

$$R_s = R_S^0 + \frac{\rho_{GB}}{l_g} \qquad (Supplementary\ Equation{:}\ 1)$$

R_s is the measured sheet resistance of the monolayer, R_S^0 is the sheet resistance of the defect free region inside a grain, ρ_{GB} is the grain boundary resistivity and l_g is the average grain size. The nucleation grain size is about 2 μm as seen in Supplementary Figure 2**b**. The grain

size of our samples was measured by analyzing SEM images just before coalescence. An image is shown in Supplementary Figure 2**c**. Line crossings were counted to determine the grain size. This was calculated to be 7.08 μm with a standard deviation of 0.88 μm. This is also in agreement with the water permeation measurements. Grain size depends on the conditions during its nucleation and growth up to 6 minutes. Since all the samples from S_1–S_4 had the same conditions, the average grain size remains approximately same. The range of sheet resistances observed in this work is shown in yellow in Supplementary Figure 2**a**. In the literature [9], the lowest sheet resistance measured across various grain sizes is taken as R_S^0. Taking R_S^0 to be 159 $\Omega\square^{-1}$ (lowest measured sheet resistance in sample S_2), a grain boundary resistivity of 3.8 kΩ-μm is calculated for the sample S_1. This grain boundary resistivity drops to 2.9 kΩ-μm and 1.7 kΩ-μm when the S_1 is annealed at 68 kJ mol^{-1} and 72 kJ mol^{-1} respectively (Samples from group G_2). The boundary resistivity in the reverse annealed sample S_3 would be like S_1.

In Supplementary Figure 2**a**, literature data shows that the sheet resistance does not vary significantly with the grain size after 1μm thereby implying that increasing grain size above 1 μm is of little value to improving the properties of the monolayer. However, the work done as part of this study show at grain sizes beyond 1 μm, grain boundaries become very important, as a large range of resistivities can be obtained depending on the degree of closure. The range of sheet resistances measured in this study is superimposed on this plot.

Supplementary Note 3 Growth setup and supersaturation calculations

Graphene growth was performed in a homemade CVD reactor equipped with gas purifiers, temperature, pressure and flow sensors. All the growth experiments were conducted at 1000°C in a horizontal tube furnace (Thermo-Fisher Lindberg BlueM) equipped with a 1 inch dimeter tube. Temperature was calibrated by using the melting point of Ge as a measure. A piece of a single crystal Ge wafer, routinely used for device fabrication in our facility, was used for this purpose. Cu foil of 99.98 % purity was obtained from Sigma-Aldrich and the sample was pre-cleaned using acetone-iso propyl alcohol-DI-water clean sequence with 5 minute exposure in each medium. The foils were blow dried and loaded into the 1 inch quartz

tube in quartz boats. Before every run, the loaded and closed quartz tube assembly, was leak tested using a He leak detector, to ensure specified leak rates below 4 x 10^{-9} cubic centimeters per second of He. The reactor temperatures were raised to the value required for growth at 4 Torr pressure under a hydrogen flow of 400 standard cubic centimeter per minute (sccm). The Cu foils were then annealed for 2 hours (unless otherwise stated) as mentioned in the main text before switching on methane flow into the reactor, from a vent line to a run line, to initiate graphene growth. The growth conditions are mentioned in the main text. The gas flow rates were first stabilized in the vent line before switching them into the run line to avoid turbulence.

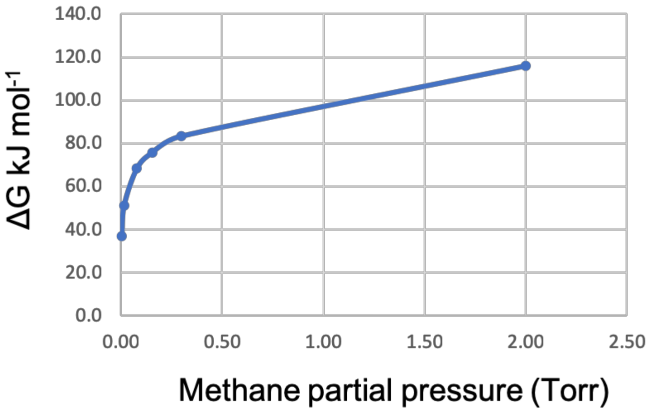

Methane flow (sccm)	Hydrogen flow (sccm)	Total pressure (P_T Torr)	P_{CH4} (Torr)	P_{H2} (Torr)	Supersaturation(*ΔG kJmol*$^{-1}$)
1	1000	4	0.01	3.99	36.67
2	500	4	0.02	3.98	51.20
10	500	4	0.08	3.92	68.19
20	500	4	0.16	3.84	75.64
40	500	4	0.30	3.70	83.28
100	100	4	2.00	2.00	116.14

Supplementary Figure 3: Thermodynamics details. Plot of supersaturation (ΔG) vs methane partial pressure P_{CH4} at a total pressure of 4 Torr and 1000°C temperature. Flow conditions are indicated in the table below. The supersaturations used in preparation of samples $S_{1\text{-}4}$ and indicated in Figure 1 of the main text during various stages of nucleation, growth and annealing of graphene were obtained from the plot above. The gas flows and corresponding supersaturation are also provided.

For the methane decomposition reaction, $\mathrm{CH_4} \rightarrow \mathrm{C_{cu}} + 2\,\mathrm{H_2}$, $\Delta G^o = 54$ kJ mol^{-1} [10] at 1000°C. The equilibrium rate constant K^{eq} given by

$$K^{eq} = \left(\frac{P_{\mathrm{CH4}}}{P_{\mathrm{H2}}^2}\right)^{eq} = \exp\left(-\frac{\Delta G^{\circ}}{RT}\right) \qquad (Supplementary\ Equation: 2)$$

can be calculated to be 0.0057 at 1000°C. P is the partial pressure of the corresponding gas

as indicated by the subscripts. The supersaturation calculated by using

$$\Delta G = RTln\left(\frac{K}{K^{eq}}\right) \qquad (Supplementary\ Equation: 3)$$

and is plotted in Supplementary Figure 3 below. K represents the experimentally imposed $\frac{P_{\mathrm{CH4}}}{P_{\mathrm{H2}}^{2}}$ ratio. Partial pressures were calculated by multiplying total reactor pressure by fractional flow rates.

Supplementary Note 4 Raman characterization of graphene films

Raman spectra was obtained using a 532 nm green laser with 1 µm spot size. The data presented in the main article is representative of a Raman map of 20 x 20 points over a 10 µm x 10 µm region. The spectra were obtained with filters to intentionally lower the laser power to <0.3 mW. This reduces the laser induced damage to the film as a single map typically takes 4-5 hours. Representative Raman spectra obtained from a single spot is shown in Supplementary Figure 4**a**. The acquired spectrum is shown in blue. Background correction was performed by using a second order polynomial fit and the corrected image is shown in orange. The peaks were then fitted with a Gaussian function. The intensity, position and width were extracted and plotted separately. The Raman maps compiled from data obtained from every spot on the three types of samples used – S_1 (6 minutes), S_2 and S_3 in this article are shown in Supplementary Figure 4**b** –**d**. The map plots the ratio of the 'D' peak intensity to the 'G' peak intensity of the Raman spectrum obtained from a 20 x 20 point array. The overall graphene defect density value for a particular sample measured by Raman spectroscopy is obtained by averaging the values obtained in the mapped region. It is to be noted that the scales, blue to yellow, are not all the same. The maps show that the I_D/I_G ratio decreases from S_1 to S_2 and S_3. Both, the large mobility difference between S_2 and S_3 (See Figure 2**b** of main text) and the trends in mobility are not captured by these Raman maps.

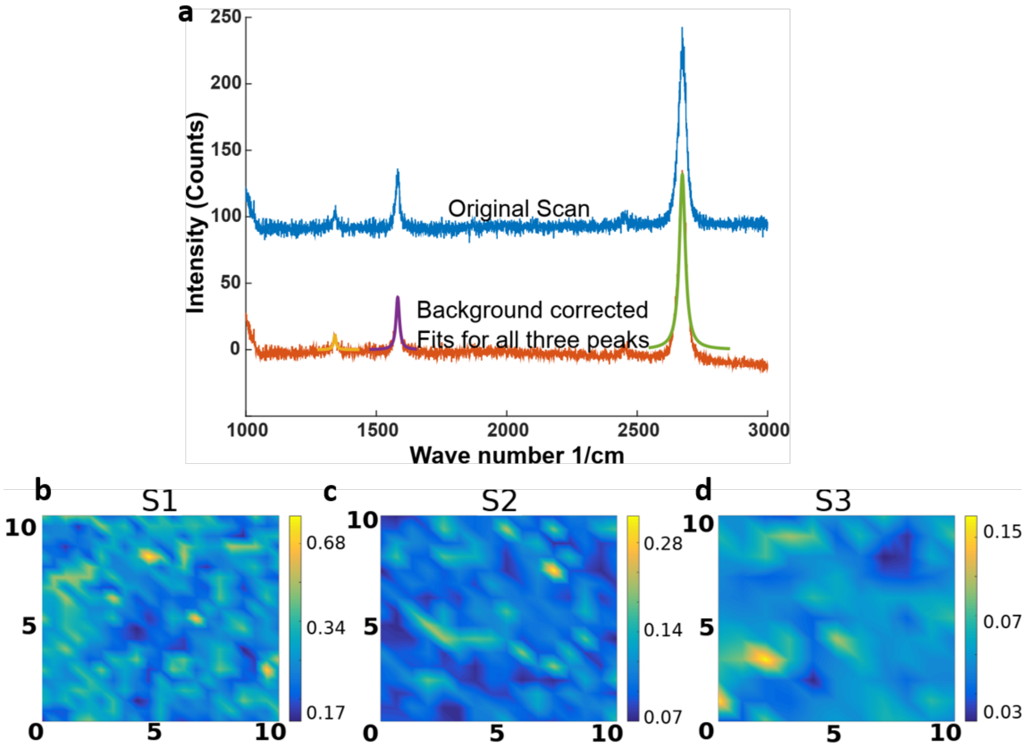

Supplementary Figure 4: Raman measurements details. **a** Raman spectra obtained at one spatial location is shown in blue. The background corrected image is shown in orange. The three most significant peaks, 'D', 'G' and '2D' were fitted with a Gaussian function. **b**–**d** Raman map of three samples S_1 (6 minutes VCG), S_2 and S_3. The map plots the ratio of the 'D' peak intensity to the 'G' peak intensity, which is a quantitative measure of the defect density in the sample. Note that the vertical scales are different.

.

Supplementary Note 5 Electrical measurements

Supplementary Figure 5**a** and 5**b** show representative images of devices used for sheet resistance (R_s) and mobility (μ_{cm}) measurement respectively. The schematics are shown in Supplementary Figure 5**c** and 5**d**. Supplementary Equations 4 – 6 were used for data analysis.

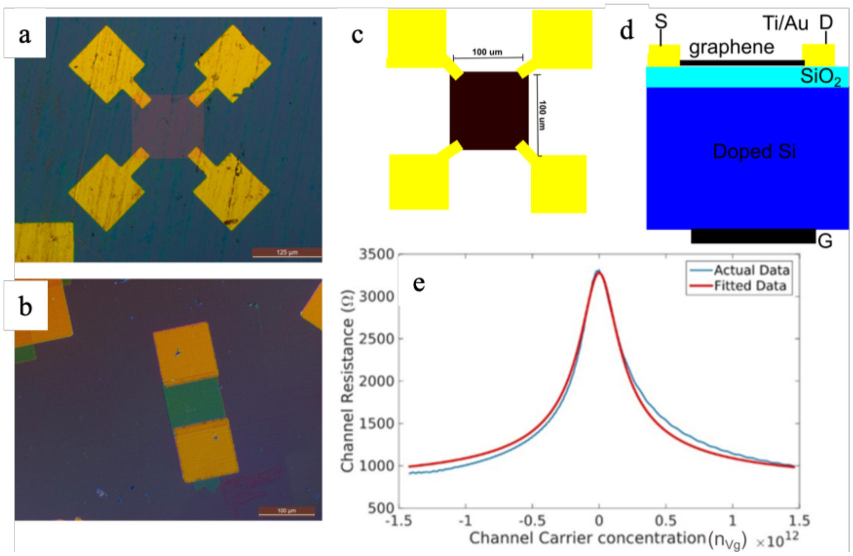

Supplementary Figure 5: **a** Contact pattern for devices used to extract sheet resistance in Van der Pauw geometry. **b** Top view of FET device used to measure transistor characteristics. **c** Schematics with device dimensions used for sheet resistance measurement. **d** Cross section of the transistor used to extract field effect mobility. Thicknesses of the oxide, Ti and Au were 285 nm, 10 nm and 100 nm respectively. **e** Experimental data and constant mobility fit.

$$R_{ch} = R_c + \frac{W}{L}\frac{1}{\mu_{cm}\sqrt{{n_0}^2 + {n_{Vg}}^2}} \qquad (Supplementary\ Equation: 4)$$

$$n_{Vg} = C_G(V_G - V_S) \qquad (Supplementary\ Equation: 5)$$

$$e^{-\frac{\pi R_{vert}}{R_S}} + e^{-\frac{\pi R_{horz}}{R_S}} = 1 \qquad (Supplementary\ Equation: 6)$$

R_{ch} is the channel resistance, W is the device width, L is the device length, n_0 is the intrinsic carrier density, n_{Vg} is the gate dependent carrier density, V_G is the gate voltage, V_S is the source voltage, C_G is the gate capacitance, R_{vert} and R_{horz} are the vertical and horizontal resistance according to Van der Pauw measurement convention. Contact resistance R_c, constant mobility μ_{cm}, and intrinsic carrier density n_0 were obtained by fitting the measured R_{ch} vs n_{Vg} data with Supplementary Equation 4. n_{Vg} was calculated using Supplementary Equation 5. A sample fit is shown in the Supplementary Figure 5**e**. The sheet resistance was extracted from measurements on a symmetric Van der Pauw structure using Supplementary Equation 6. The fit values so obtained for the samples described in the manuscript are given

in the table below.

Supplementary Table 1: List of samples and fit values obtained for the samples discussed in the main text. The group in column 1 corresponds to the group in Table 1 of the main manuscript. R_c is the contact resistance, n_o is the intrinsic carrier density, I_d/I_g is the defect density value and std. err. is the standard deviation in the measurements. Row number 5, 14, 18 and 19 correspond to samples S_1, S_2, S_3 and S_4 respectively

Group	Row No.	Growth condition	R_c (Ω)	n_o ($\times10^{11}$ cm^{-2})	μ_{cm} (cm^{2}V^{-1}s^{-1})	μ_{cm}_err (cm^{2}V^{-1}s^{-1})	ID/IG	ID/IG err	Rs ($\Omega\square^{-1}$)	Rs_err ($\Omega\square^{-1}$)
	Standard samples: Shown in Figure 1(**c** and **d**)									
G_1	1	ΔG = 36 kJ mol-1, 2.5 min	1503	5.1	210	10	1.2	1	7192	1890
	2	ΔG = 36 kJ mol-1, 3 min	1876	11.42	234	21	1.35	1.2	1576	580
	3	ΔG = 36 kJ mol-1, 4 min	2080	6.18	400	219	1.11	0.8	1743	620
	4	ΔG = 36 kJ mol-1, 5 min	1489	3.2	505	320	0.8	0.42	1724	885
	5	ΔG = 36 kJ mol-1, 6 min	2669	32.5	980	130	0.32	0.21	1560	945
	6	ΔG = 36 kJ mol-1, 7 min	510	12.35	2875	350	0.22	0.08	1022	840
	7	ΔG = 36 kJ mol-1, 10 min	1761	14.6	3115	890	0.133	0.05	944	570
	8	ΔG = 36 kJ mol-1, 12 min	1917	5.58	3479	379	0.154	0.03	802	384
	9	ΔG = 36 kJ mol-1, 13 min	1973	22.61	3667	987	0.16	0.07	741	275
	10	ΔG = 36 kJ mol-1, 14 min	1594	11.86	3687	280	0.16	0.06	763	243
	Annealed graphene: Shown in Figure 2**a**									
G_2	11	S1 + ΔG = 36 kJ mol-1, 6min	439	11.80	3182	187.5	0.123	0.03	619	54
	12	S1 + ΔG = 68 kJ mol-1, 6 min	698	0.00	4982	354	0.139	0.02	579	55
	13	S1 + ΔG = 74 kJ mol-1, 6 min	703	2.00	8877	987	0.159	0.02	450	29
	14	S1 + ΔG = 82 kJ mol-1, 6 min	1323	1.20	19862	2120	0.158	0.03	218	44
	15	S1 + ΔG = 115kJ mol-1, 6 min	1761	0.86	15687	5182	0.151	0.02	175	56
	Reverse annealed graphene: Shown in Figure 2**b**									
G_3	16	S2 + ΔG = 74J/mol, 4 min	408	2.16	12239	2556	0.156	0.02	466	48
	17	S2 + ΔG = 68kJ mol-1, 4 min	2044	1.00	7704	754	0.123	0.01	642	56
	18	S2 + ΔG = 36kJ mol-1, 4 min	361	0.90	6821	465	0.191	0.02	726	72
	Hydrogen treated graphene									
G_4	19	S2 + H2 treatment for 4 mins	121	4.20	12000	1564	0.219	0.05	210	32
	Graphene samples for Figure 5									
	20	ΔG = 36kJ mol-1, 10 min	1653	11.80	3182	187.5	0.123	0.03	619	54
	21	ΔG = 50kJ mol-1, 10 min	1557	10.00	2989	156	0.131	0.01	4851	387
	22	ΔG = 82kJ mol-1, 10 min	1244	6.00	2678	432	0.07	0.02	6495	632
	23	ΔG = 36kJ mol-1, 10 min + 5 mins H2	2157	11.00	2875	287	0.14		1169	158
	24	ΔG = 50kJ mol-1, 10 min + 5 mins H2	1687	10.00	1990	166.7	0.114	0.01	9751	874
	25	ΔG = 82 kJ mol-1, 10 min + 5 min H2	804	11.00	1217	100.3	0.15	0.05	12895	980
		Samples for repeatability tests: Supplementary Figure 5-2**b** Sample S_2								
	26	S1 + ΔG = 82 kJ mol-1, 6 min	1323	1.20	19862	2120	0.158	0.03	218	44
	27	S1 + ΔG = 82 kJ mol-1, 6 min	1178	0.42	20687	989	0.182	0.1	155	23
	28	S1 + ΔG = 82 kJ mol-1, 6 min	2550	1.67	20058	2182	0.143	0.01	159	12

Supplementary 6 Electrical measurement statistics and fitting

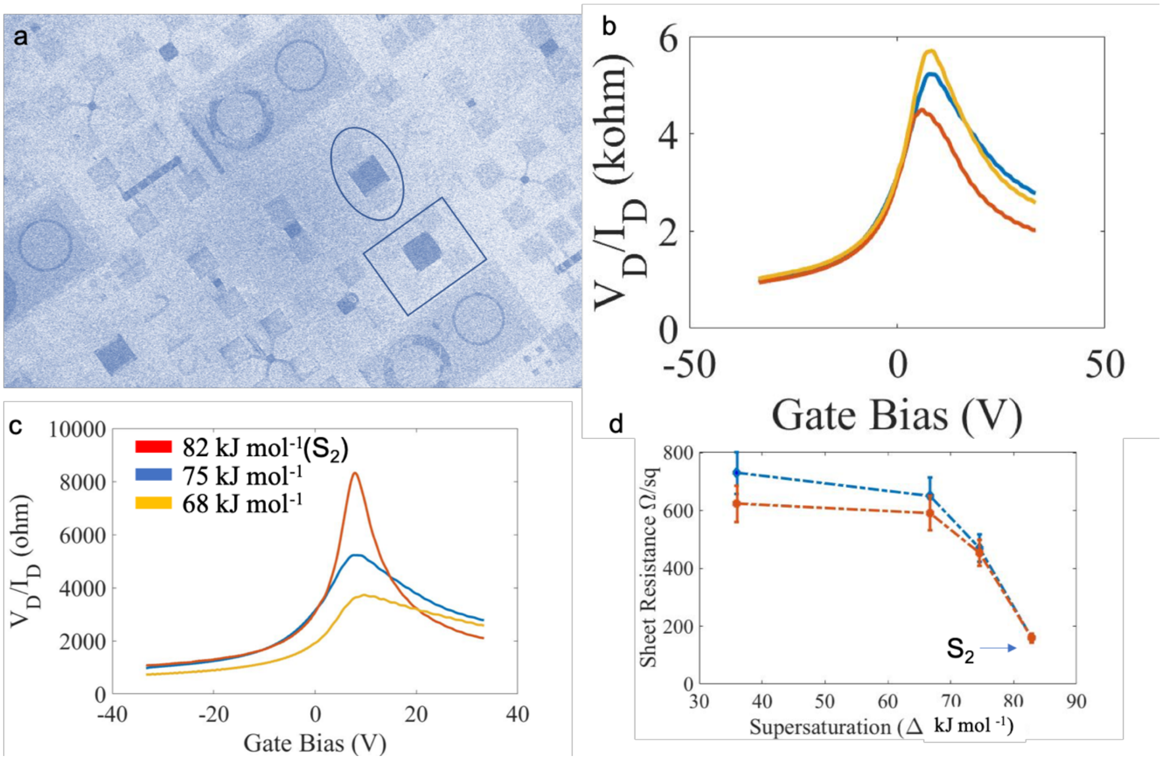

Supplementary Figure 6: **a** Device array to show various kinds of structures such as Van der Pauw and FET structures, which are indicated by a square and ellipse respectively. **b** Channel resistance vs gate bias for three different growth trials of sample S_2 under the same condition (as mention in the table 1 row numbers 26 – 28). **c** Channel resistance vs gate bias for the three samples that make up Supplementary Figure 2**a**. It can be easily seen that the intrinsic carrier density drops as the mobility increases. **d** The plot of sheet resistance vs ΔG showing decreasing trend with a minimum value of 159 ohm$□^{-1}$ achieved.

An array of fabricated devices is shown in Supplementary Figure 6**a**. For each graphene growth, at least 20 devices with Van der Pauw structures and FET devices were fabricated. We measured at least 4 devices for statistical purposes. We have also repeated the growth for reliability tests. Supplementary Figure 6**b** shows the channel resistance vs gate bias for sample S_2 for three different growth trials (see table 1 rows 26 – 28). Supplementary Figure 6**c** shows the channel resistance vs gate bias for three samples whose mobilities are plotted in Figure 2**a** of the main text. These three samples were obtained by taking S_1(VCG) and annealing them at the higher methane partial pressures indicated. The 82 kJ mol^{-1} is sample S_2. The increase in mobility with supersaturation can be easily noted from the change in slope. It is also important to note that the unintentional doping density reduces as the mobility increases. This can be inferred from the shift in the zero gate bias towards zero voltage as

well as increase in the zero bias resistance. Thus, the reduction in sheet resistance, Supplementary Figure 6**d**, is due to the rise in mobility and not due to doping. Rather, it is due to a reduction in defect density at the grain boundaries.

Supplementary Note 7 Device fabrication for graphene films before coalescence

At a growth time of less than 6 minutes the monolayers are incomplete. There are however areas in which patches of graphene monolayers with grain boundaries are obtained. These areas allow for testing of the electrical characteristics of the boundaries, albeit by using device channel dimensions that are smaller than 100 μm. Electron-beam lithography was used to pattern devices in such areas. Two such images are shown in the Supplementary Figure 7 below. Typical channel dimensions in these samples were from 5 μm to 15 μm depending on the size of the coalesced areas available.

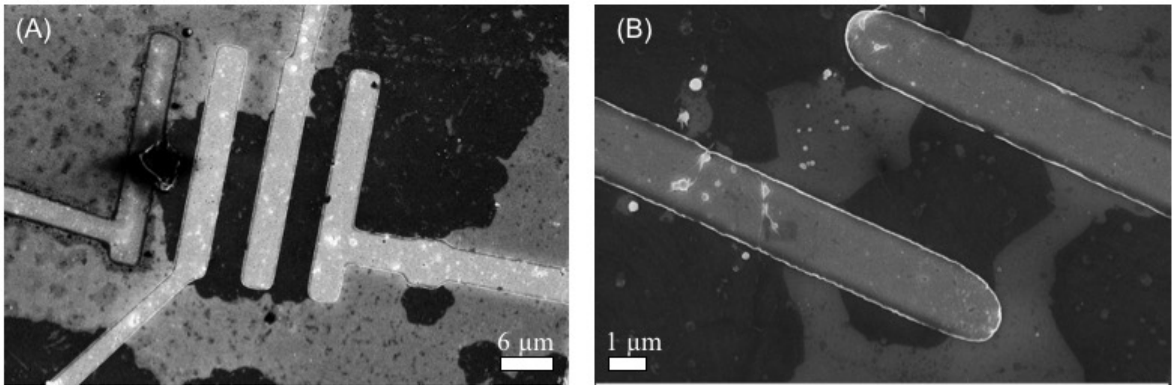

Supplementary Figure 7: **a** Electron beam lithography fabricated device for electrical measurements before complete coalescence. **b** Fabricated electrical contacts for two grains that have merged.

Supplementary Note 8 DFT computation details:

The reaction scheme used in DFT to model our CVD process is summarized in the 9 reactions below.

$GB + \frac{1}{2} n_1 H_2 \rightarrow GE\text{-}H + n_1 C$ (graphite) (*Supplementary Equation*: *7)*

n_1C(graphite)$+2n_1 H_2 \rightarrow n_1CH_4$ (*Supplementary Equation*: 8)

n_2C (atoms) $\rightarrow GE$ (*Supplementary Equation*: *9)*

n_3C (atoms) $\rightarrow GB$ (*Supplementary Equation*: *10)*

n_1C(atoms) $\rightarrow n_1C$(graphite) (*Supplementary Equation*: *11)*

n_1H (atoms) + GE → GE-H (*Supplementary Equation*: *12)*

n_1H (atoms) → ½ n_1 H_2 (*Supplementary Equation*: *13)*

$n_1 + n_2 \rightarrow n_3$ (*Supplementary Equation*: *14)*

The actual reaction during growth is the sum of Supplementary Equations (*7)* and (*8*) the way we see it in our scheme. Its energy change, $\Delta G = G_{\text{Reactants}}\text{-}G_{\text{Products}}$, can be calculated as summed below. This energy change is what is plotted in Supplementary Figure 4. Positive changes favor the forward reaction and negative changes the reverse reaction.

GE-H + n_1CH_4 → GB + 2½ n_1 H_2 (*Supplementary Equation*: *15)*

$\Delta G_9 = \Delta G_1 + \Delta G_2 \rightarrow (\Delta G_3 - \Delta G_4 + \Delta G_5 + \Delta G_6 - \Delta G_7) + \Delta G_2$

In contrast, the energy change by Dong et al. [11] is for

GE-H → GB + H (*Supplementary Equation*: *16*

All first principles calculations in this work were performed using plane wave basis as implemented in Quantum Espresso [12] package. We use norm-conserving pseudopotentials [13] and generalized gradient approximation (GGA) [14] for the exchange-correlation functional. The wavefunctions in our calculation were expanded using plane waves up to a cutoff energy of 70 Ry. We found this to be sufficient to ensure the numerical error of the results to be less than 1 meV/atom. The lattice constants as well as the atomic positions in all the structures were relaxed using Broyden-Fletcher-Goldfarb-Shanno (BFGS) quasi-newton algorithm, such that the pressure on the unit cell was less than 0.5 Kbar and forces on each atom were less than 10^{-3} Ry/a.u. For the first grain boundary (GB1) we sampled the Brillouin zone with only Γ point because of the large supercell required to model it. For the second grain boundary calculation we sampled the Brillouin zone with two k-points.

We study two model grain boundaries, namely GB1 and GB2. In figure 4 of the main article we show the schematic of these GBs. As we can see from figure 6**m** – **o** , GB1 is much wider than GB2, but GB1 has a misorientation angle of ~13° [15]. The aim of this study was to understand the effect of environmental conditions (during the growth of graphene) on GB formation. For both the GBs (GB1 and GB2) we performed two different calculations. First, we calculated the total energy of the supercell that contained the respective GB structure (E_{GB}). In the next phase of the calculation we removed carbon atoms from the GB region. Upon removing carbon atoms from the GB region, we formed two grain edges (GE), which

were then passivated with hydrogen atoms. The exact structures studied are shown in figures 4**a** and figure 4**b** in the main article. We calculated the total energy of this new supercell containing two GEs (E_{GE}). Now if we consider following chemical reaction,

$$GB + \frac{1}{2} n_l H_2 = GE\text{-}H + n_l C \text{ (graphite)} \qquad (\textit{Supplementary Equation: 17})$$

where GE structure and some additional (n_C) carbon atoms combine to form a GB structure. The n_l hydrogen atoms that were passivating the GEs evolve as ½ n_l hydrogen molecules. One can compute the change in Gibbs free energy of this reaction (GB formation energy from two GEs) as

$$\Delta G [P, T] = E_{GB} - E_{GE} - n_C \mu_C[P, T] + n_H \mu_H[P, T] \qquad (\textit{Supplementary Equation: 18})$$

where E_{GB} and E_{GE} are the total energies of the supercells containing GB and GE structures respectively. We obtained these total energies from first principles calculations and assumed them to have a negligible temperature pressure dependence. $\mu_C[P, T]$ and $\mu_H[P, T]$ are the chemical potentials of carbon and hydrogen atoms. As we are interested in formation of graphene sheets from methane, we chose the chemical potential of carbon atom in a CH_4 molecule as our μ_C. μ_H has been set to chemical potential of hydrogen atom in a hydrogen molecule. This is because the H atoms that are released from the surface go into the chamber as H_2 gas. If one considers following chemical reaction where graphite reacts with hydrogen to form methane,

$$C_{graphite} + 2H_2 \rightarrow CH_4 \qquad (\textit{Supplementary Equation: 19})$$

temperature dependent chemical potentials of carbon atom in CH_4 molecule ($\mu_C[CH_4, P, T]$) can be written as,

$$\mu_C[CH_4, P, T] = \mu_C[\text{graphite}] + \Delta G[CH_4, P, T] \qquad (\textit{Supplementary Equation: 20})$$

where μ_C[graphite] is the chemical potential of carbon atom in graphite at T, P=0, which can be obtained from first principles calculation. $\Delta G[CH_4, P, T]$ is the Gibbs free energy of formation for methane as a function of temperature and pressure which we obtain from JANAF thermochemical table [16]. While JANAF table provides the values at 1atm pressure, we assumed ideal gas behavior to obtain the value at other pressures. On the other hand, the chemical potential of hydrogen atom in H_2 molecule ($\mu_H[H_2, P, T]$) can be obtained using the following Supplementary Equation,

$$\mu_H[H_2, P, T] = \mu_H[H_2] + \frac{1}{2} \Delta H[P, T] \qquad (\textit{Supplementary Equation: 21})$$

where $\mu_H[H_2]$ is the chemical potential of hydrogen atom in H_2 molecule at T=0 (which is

obtained from first principles calculation) and $\Delta H[P, T]$ is the enthalpy of a H_2 molecule relative to its T=0 value, which also can be obtained from JANAF thermochemical table (We have to keep in mind that in the JANAF tables $\Delta H[T]$ is listed relative to 298.15° K value but we use 0° K value as our reference). Inclusion of zero-point corrections are crucial to obtain the experimentally observed temperature for the decomposition of methane. As a result, we have included zero-point energy corrections (from NIST CCCBD tables) in case of total energy calculation for methane and hydrogen molecule.

The free energy change for the Supplementary Equation 15 at 1000°C, calculated by the procedure just describe, as a function of methane partial pressure is plotted in Figure 4 of the main text.

Supplementary Note 9 Effect of supersaturation on graphene grain size

For the results shown in figure 5 of the main manuscript, graphene monolayers were

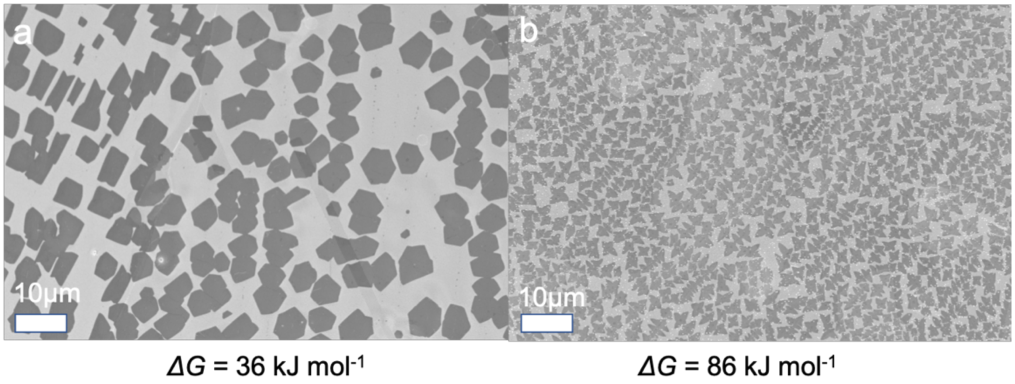

Supplementary Figure 8: Effect of supersaturation **a** 36 kJ mol^{-1} and **b** 82 kJ mol^{-1} at nucleation on grain size

nucleated and grown at different ΔGs to obtain different grain sizes. The set of experiments were conducted to quickly prove that the degradation happens at grain boundaries and triple junctions. Supplementary Figures 8**a** and **b** show that increasing supersaturation from 36 and 82 kJ mol^{-1} during nucleation, would decrease grain size in the coalesced monolayer. This in turn would increase the grain boundary length per unit area. The fact that sheet resistance degradation increases with a decrease in grain size shows that the responsible defects are associated with the grain boundaries.

Supplementary Note 10 Ge etch experiments:

The defect structures in graphene were made visible using a technique reported by Suran et al. [17]. A drop of water is placed on a graphene monolayer transferred on to a Ge film. Water percolating through defects in the graphene layer oxidizes and dissolves the Ge below. The

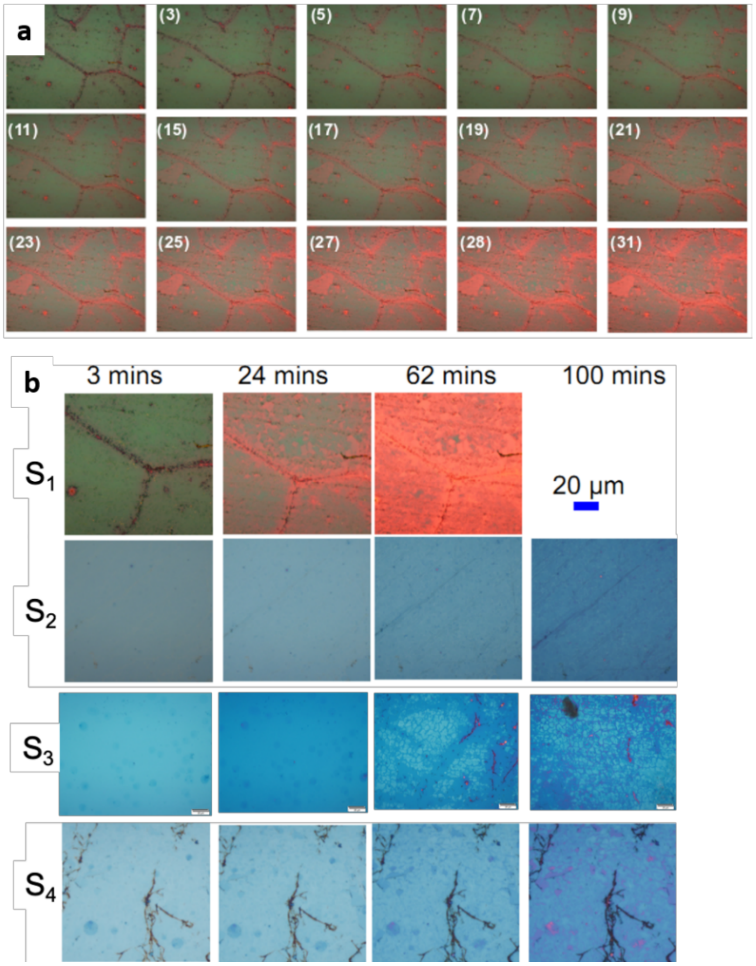

Supplementary Figure 9: Etch sequence comparison: **a** Revealing defects in a graphene monolayer by studying the etching of an underlying Ge film: The optical microscope snapshot images of Graphene-on-Ge surface is shown after exposure to water with time in minutes is shown in each image. Preferential permeation of water is seen as the color of certain regions start to change from green to red. The line features seen in this time sequence of VCG represent grain boundaries in the Cu layer used for deposition. They show that in VCG graphene grown on top of the Cu grain boundaries are seriously defective as discussed in the main paper. **b** The Ge etch experiments were conducted on four graphene coated films. The micron markers are all 20 μm. The etch sequence comparison for the 2 hours annealed Cu foil is shown for the four samples S_1, S_2, S_3 and S_4 taken at 3 minutes, 24 minutes, 62 minutes and 100 minutes respectively. The etch sequence observed in each of the samples are presented row-wise. The etch in the case of S_1 was stopped at 62 minutes because the Ge film was almost etched, and the differences ceased to be prominent. The etch was predominantly happening in the sample S_1 along the Cu grain boundaries making them more visible than the rest of the regions. In S_2 and S_3, the etch rate was lower and was predominantly happening at the graphene grain boundaries

etch profile thus created is a replica of the microstructure of graphene layer. A sample time

sequence of the etching that reveals the graphene microstructure is shown in Supplementary Figure 9.

Supplementary Note 11 AFM scans of etch pits

The AFM scan images of the etch pits in two samples (S_2 and S_3), are shown in Supplementary Figure 10 at 100 minutes. In S_1 and S_4 in which rather rapid etching was observed in under 60 minutes, the rapid etch rates prevent reliable extraction of etch dimensions. The typical etch pit width in samples S_2 and S_3 were 56 nm and 474 nm respectively. The large area scan shown in Supplementary Figures 10**a** and **c** clearly show the impact of reverse annealing on the permeation of graphene grain boundaries. The width dimension in a representative boundary is shown for each case in Supplementary Figures 10**b** and **d**.

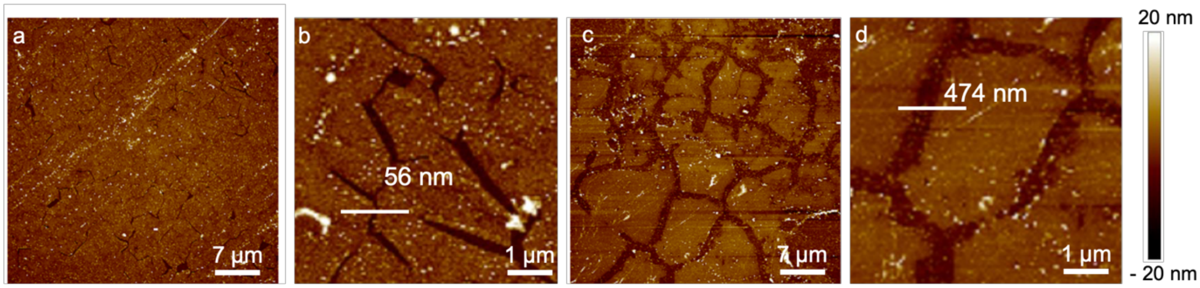

Supplementary Figure 10: Etch depth analysis. AFM scans showing grain boundary etching in two samples S_2, and S_3 are given. **a** Large area scan of sample S_2 and **b** shows the magnified image with line scan showing the pit width of 56 nm. **c** Large area scan of sample S_3 and **d** shows the magnified image with line scan showing the pit width of 474 nm. The scale bar indicates the etch depth in each of the samples indicating the etch rate. Sample S_2, which underwent annealing under higher ΔG had lower etch rates indicating a boundary formed with lower defect density.

Supplementary Note 12 TEM tilt measurements for monolayer coverage

Monolayers can be identified in TEM as described by Meyer et al.[18] Diffraction patterns taken at increasing tilt angles having monotonous change in the intensity indicate the presence of monolayer graphene. Below one such tilt sequence is shown. It is clearly seen in Supplementary Figure 11 that until a tilt of 10°, there is a monotonous decrease in the diffraction spot intensity. In predominantly bilayer graphene cyclical changes to the intensity would be observed.

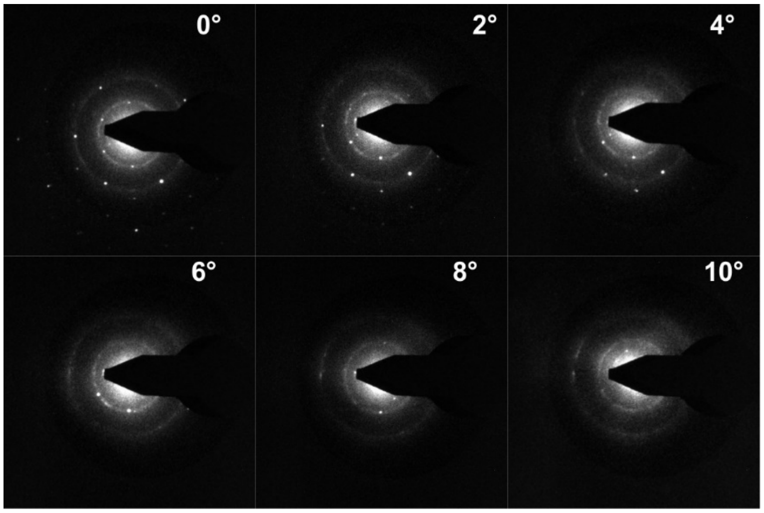

Supplementary Figure 11: Diffraction measurements. Diffraction images taken at various tilt angles of the sample. The spot intensity undergoes a monotonic change in FWHM and intensity showing that the sample is indeed a monolayer.

Supplementary Note 13 Angular Misorientation of grains by TEM

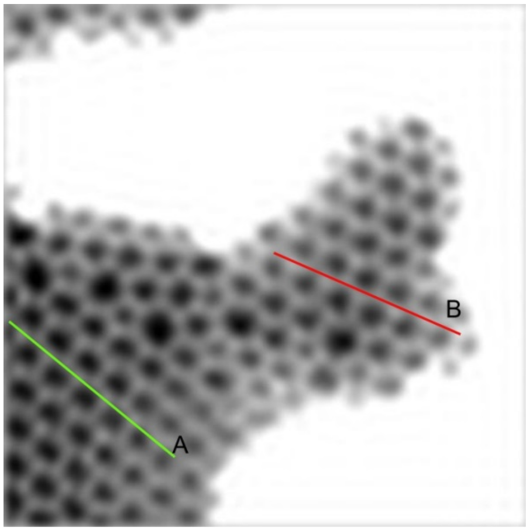

Supplementary Figure 12: Grain misorientation measurement. Two lines, shown, were used to measure the angular misorientation between the grains.

The angular misorientation between the grains shown in main article was calculated using ImageJ angle measurement software. The two lines used for angle calculation are shown in Supplementary Figure 12. An angular misorientation of 13.8° was measured. Simulations

made by Yazyev et al. [15, 19, 20] were used to determine the hexagon to Stone-Wales defect site ratio.

A representative image of the calculated number of 5-7 structures to the standard hexagons are shown in Supplementary Figure 13**a**. At least two rows of hexagons were first established to identify the grains and the angle between them. This has been marked by the white lines in Supplementary Figure 13**b** below. Once the two rows were identified, the region in between was assumed to constitute the grain boundary. It is made up of hexagons and pentagons and heptagons whose numbers were determined to be 13, 8 and 8 respectively. The ratio (8*2/13) is greater than 1.

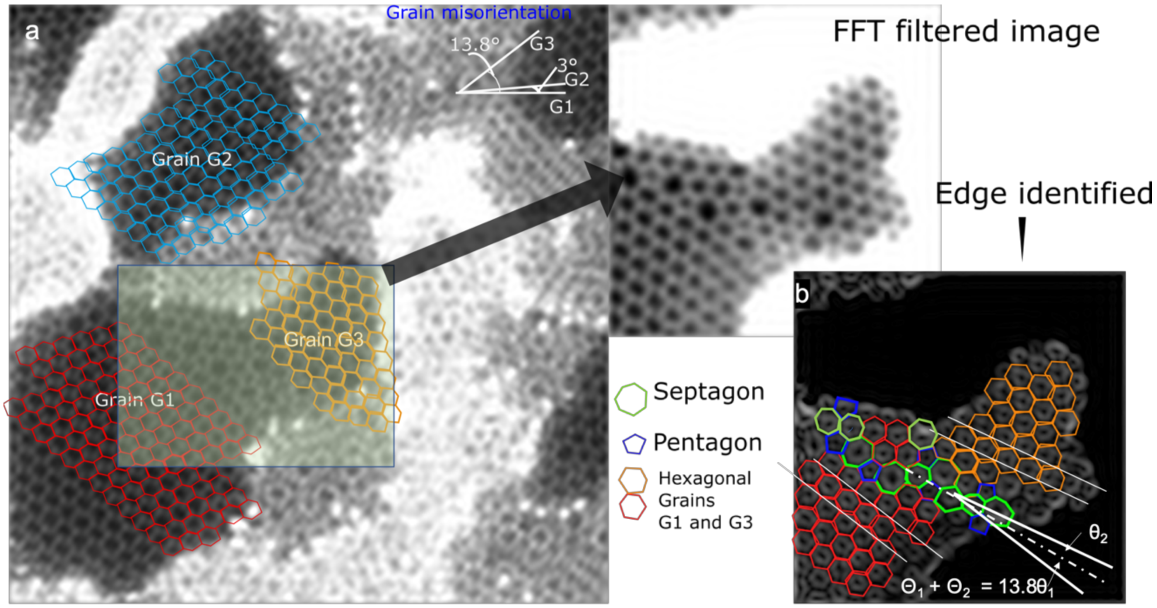

Supplementary Figure 13: TEM image analysis. **a** Grain boundary image using HRTEM. **b** The angular misorientation of the grains were determined and observed to be symmetric with respect to the boundary defects. The hexagonal graphene lattice and the defect structures between the grain is identified. The ratio of the hexagon to 5-7 structures was calculated as detailed above.

Supplementary Note 14 Misorientation defect density calculations

The angular misorientation of grains G1 and G3 with respect to the grain boundary (dotted line), denoted by θ_1 and θ_2, is shown in Supplementary Figure 13**b**. The total misorientation $\theta = \theta_1 + \theta_2$ is 13.8 degrees and it is almost a symmetric boundary. From Yazyev and Louie [19] the grain boundary energy, is seen to be less than 0.50 eV/Å for a symmetric 14° misorientation.

Taking the formation energy of hexagon lattice as reference, the formation energy of Stone-Wales defect is reported to be about 7.5 eV [19]. As shown in Supplementary Figure 14, using the C=C bond length of 1.42 Å, the Stone-Wales heptagon-pentagon pair (vertex to vertex) length can be calculated to be $(C_b/2*(1+\sqrt{5}) + C_b / (2 * \tan(\pi/2/7)))$ 5.3 Å and that of hexagon-hexagon pair to be $3*C_b$ or 4.26 Å. Given these numbers, if "x" is the number of Stone-Wales defects and "y" is the number of hexagon pairs, the energy per unit length of the grain boundary can be calculated using the left side of Supplementary Equation (22). When, this is equated to the number 0.5 eV/ Å, the boundary energy [19], the ratio (x/y) can be calculated to be 0.46.

$$\frac{7.5 * x}{5.3 * x + 4.26 * y} = 0.5 \qquad (Supplementary\ Equation: 22)$$

The number at the boundaries observed by us is greater than 1. The triple junction which is close to the imaged boundary could also influence the defect density. However, what this simple calculation nevertheless shows is that defect densities at boundaries and especially closer to triple junctions would be much larger than those theoretically anticipated. They, in turn, could have a significant impact on the properties of the monolayer.

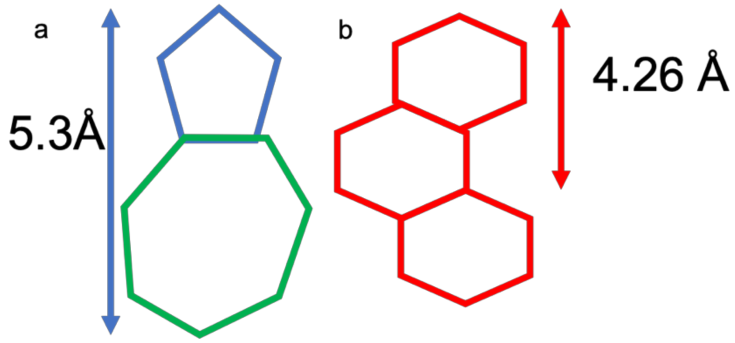

Supplementary Figure 14: Defect structures. Calculated length for **a** Stone-Wales defect site and **b** hexagon-hexagon pair.

Supplementary References

1. Yan, Z., Lin, J., Peng, Z., Sun, Z., Zhu, Y., Li, L., Xiang, C., Samuel, E. L., Kittrell, C. & Tour, J. M., Toward the Synthesis of Wafer-Scale Single-Crystal Graphene on Copper Foils. *ACS Nano* **6** (10) (2006).
2. Wang, D., Liu, X., He, L., Yin, Y., Wu, D. & Shi, J., Manipulating graphene mobility and charge neutral point with ligand-bound nanoparticles as charge reservoir. *Nano lett.* **10**, 4989-4993 (2010).
3. Yang, Y. & Murali, R., Impact of size effect on graphene nanoribbon transport. *IEEE Electr. Device L.* **31**, 237-239 (2010).
4. Gao, L., Ren, W., Xu, H., Jin, L., Wang, Z., Ma, T., Ma, L.-P., Zhang, Z., Fu, Q., Peng, L.-M., Bao, X. & Cheng, H.-M., Repeated growth and bubbling transfer of graphene with millimetre-size single-crystal grains using platinum. *Nat. Comm.* **3**, 699 (2012).
5. Li, X., Cai, W., An, J., Kim, S., Nah, J., Yang, D., Piner, R., Velamakanni, A., Jung, I., Tutuc, E. & others, Large-area synthesis of high-quality and uniform graphene films on copper foils. *Science* **324**, 1312-1314 (2009).
6. Buron, J. D., Pizzocchero, F., Jepsen, P. U., Petersen, D. H., Caridad, J. M., Jessen, B. S., Booth, T. J. & B, Graphene mobility mapping. *Sci. rep.* **5**, 12305 (2015).
7. Banhart, F., Kotakoski, J. & Krasheninnikov, A. V., Structural defects in graphene. *ACS nano* **5**, 26-41 (2010).
8. Banszerus, L., Schmitz, M., Engels, S., Dauber, J., Oellers, M., Haupt, F., Watanabe, K., Taniguchi, T., Beschoten, B. & Stampfer, C., Ultrahigh-mobility graphene devices from chemical vapor deposition on reusable copper. *Sci. adv.* **1**, e1500222 (2015).
9. Isacsson, A., Cummings, A. W., Colombo, L., Colombo, L., Kinaret, J. M. & Roche, S., Scaling properties of polycrystalline graphene: a review. *2D Mat.* **4**, 012002 (2016).
10. Ghosh, P., Kumar, S., Ramalingam, G., Kochat, V., Radhakrishnan, M., Dhar, S., Suwas, S., Ghosh, A., Narayanan, R. & Raghavan, S., Insights on Defect-Mediated Heterogeneous Nucleation of Graphene on Copper. *J. Phys. Chem. C* **119** (5), 2513 - 2522 (2015).

11. Dong, J., Wang, H., Peng, H., Liu, Z., Zhang, K. & Ding, F., Formation mechanism of overlapping grain boundaries in graphene chemical vapor deposition growth. *Chem. sci.* **8**, 2209-2214 (2017).

12. Giannozzi, P., Baroni, S., Bonini, N., Calandra, M., Car, R., Cavazzoni, C., Ceresoli, D., Chiarotti, G. L., Cococcioni, M., Dabo, I. & others, QUANTUM ESPRESSO: a modular and open-source software project for quantum simulations of materials. *J. Phys. Condens. Matter* **21**, 395502 (2009).

13. Troullier, N. & Martins, J. L., Efficient pseudopotentials for plane-wave calculations. *Phys. Rev. B* **43**, 1993 (1991).

14. Perdew, J. P., Burke, K. & Ernzerhof, M., Generalized gradient approximation made simple. *Phy. Rev.lett.* **77**, 3865 (1996).

15. Yazyev, O. V. & Louie, S. G., Electronic transport in polycrystalline graphene. *Nat. mat.* **9**, 806 (2010).

16. Stull, D. R. & Prophet, H., Tech. rep., 1971.

17. Suran, S., Bharadwaj, K., Raghavan, S. & Varma, M. M., Bright-field Nanoscopy: Visualizing Nano-structures with Localized Optical Contrast Using a Conventional Microscope. *Sci. rep.* **6**, 25011 (2016).

18. Meyer, J. C., Geim, A. K., Katsnelson, M. I., Novoselov, K. S., Booth, T. J. & Roth, S., The structure of suspended graphene sheets. *Nature* **446** (2007).

19. Yazyev, O. V. & Louie, S. G., Topological defects in graphene: Dislocations and grain boundaries. *Phy. Rev. B* **81**, 195420 (2010).

20. Yazyev, O. V., Polycrystalline graphene: Atomic structure, energetics and transport properties. *Solid state Commun.* **152** (15), 1431 (2012).
